# S9. RESTING EEG CHANGES IN SCHIZOPHRENIA

**DOI:** 10.1093/schbul/sby018.796

**Published:** 2018-04-01

**Authors:** Leonhardt Unruh, Rick Adams, Alan Anticevic, L Elliot Hong

**Affiliations:** 1 University of Göttingen; 2 University College London; 3 Yale University; 4 University of Maryl

## Abstract

**Background:**

Numerous previous studies have found increased power in low frequencies in resting EEG data in subjects with a diagnosis of schizophrenia (Scz). Low frequency power in δ is usually localised to frontal channels, whereas increases in θ power can be more widespread (Boutros et al., 2008). Given the low spatial resolution of EEG data, we used a spatial filter (the surface Laplacian) to investigate whether differences in resting EEG frequency band power in Scz are truly distributed throughout the cortex, or whether they localise to more focal areas.

**Methods:**

64 channel EEG data were recorded for 5 minutes in both eyes closed and eyes open conditions in 103 medicated Scz (M=71, F=32, mean(std) age=40(14.3) yrs) and 104 controls (M=64, F=30, mean(std) age=40(13.8) yrs). The data were epoched and epochs with artefacts were detected by their amplitude, variance or kurtosis and removed. The power spectral density at each channel was computed using Welch’s method, and the surface Laplacian (using the spherical spline method of Perrin et al. (1987, 1989)) was applied to reduce volume conduction effects and improve topographical localization. Power was analysed separately for five frequency bands: δ (2–4 Hz), θ (4–8 Hz), α (8–12 Hz), β (15–30 Hz) and γ (30–50 Hz). Permutation testing (20 runs of 1000 group label permutations) was performed with correction for multiple comparisons based on clusters of channels of significantly different (p<0.05 uncorrected) power.

**Results:**

Scz and controls showed group differences in θ power (mean(std) over all channels, eyes open condition: Scz=5.7(10)x104 μVmm-2 and controls=3.2(5.9)x104 μVmm-2, t test, t(181) = 2.0, p = 0.047; and at trend level significance in the eyes closed condition: Scz=6.0(9.6)x104 μVmm-2 and controls=3.8(6.2)x104 μVmm-2, t test, t(181) = 1.8, p = 0.067), but no group differences in the other four frequency bands (all p >0.05). Scz had greater θ power in both eyes open and eyes closed conditions using cluster-based permutation tests (both p<0.05). The median cluster sizes (25% quantile, 75% quantile) with elevated θ power in Scz were of 53 (51, 55) channels in the eyes open and 50.5 (48, 51.5) in the eyes closed condition.

**Discussion:**

These initial results support previous findings of widespread increased θ band power in Scz (e.g. Narayanan et al., 2014). These θ differences appear to be manifest in the majority of the cortex, rather than being localised to one particular area.

